# Complete Genome Sequence of a Polypropylene Glycol-Degrading Strain, *Microbacterium* sp. No. 7

**DOI:** 10.1128/genomeA.01400-15

**Published:** 2015-12-10

**Authors:** Yoshiyuki Ohtsubo, Yuji Nagata, Mitsuru Numata, Kieko Tsuchikane, Akira Hosoyama, Atsushi Yamazoe, Masataka Tsuda, Nobuyuki Fujita, Fusako Kawai

**Affiliations:** a Department of Environmental Life Sciences, Graduate School of Life Sciences, Tohoku University, Sendai, Japan; b Biological Resource Center, National Institute of Technology and Evaluation, Tokyo, Japan; c Center for Fiber and Textile Science, Kyoto Institute of Technology, Kyoto, Japan

## Abstract

*Microbacterium* (formerly *Corynebacterium*) sp. No. 7 was isolated from activated sludge as a polypropylene glycol (PPG)-assimilating bacterial strain. Its oxidative PPG degradation has been proposed on the basis of PPG dehydrogenase activity and the metabolic products. Here, we report the complete genome sequence of *Microbacterium* sp. No. 7. The genome of the strain No. 7 is composed of a 4,599,046-bp circular chromosome and two linear plasmids. The whole finishing was conducted *in silico* with aids of the computational tools GenoFinisher and AceFileViewer. Strain No. 7 is available from the Biological Resource Center, National Institute of Technology and Evaluation (NITE) (Tokyo, Japan).

## GENOME ANNOUNCEMENT

*Microbacterium* sp. No. 7 was first isolated as a polypropylene glycol (PPG)-utilizing strain from activated sludge acclimated to PPG, which was identified as *Corynebacterium* sp. No. 7 (1). Later, it was misidentified as *Stenotrophomonas maltophilia*, based on a commercial analysis of 16S rRNA genes (not deposited) (2). Most recently, we reidentified it as *Microbacterium* sp., as its 16S rRNA gene (accession no. LC003038) showed the closest similarity (98.2% identical) to that of *Microbacterium ulmi* (accession no. AY062021). Afterwards, we isolated several PPG-utilizing sphingomonads (3). The oxidation pathway of PPG has been proposed on the basis of PPG dehydrogenase activity and characterization of the metabolic products (4), and matrix-assisted laser desorption ionization–time of flight (MALDI-TOF) mass spectrometry confirmed that the secondary alcohol is preferentially oxidized to the keto form (5). Strain No. 7 possessed several PPG dehydrogenases (2, 6, 7), which were classified by cellular localization and electron acceptors, such as pyrroloquinoline quinone, NAD, and artificial acceptors.

The strain No. 7 genome was sequenced using 454 GS-FLX Titanium (Roche) and the HiSeq and MiSeq systems (Illumina). A fragment library was constructed for the 454 GS-FLX sequencing, and mate-pair and paired-end libraries were constructed for Illumina sequencing. The mate-pair library was sequenced by MiSeq for 251 bp apiece from both ends to obtain 2.2 M pairs (4.4 M reads). Each pair of reads was processed by ShortReadManager (SRM) to extract mate pairs (MP), while read sequences were trimmed for adaptors and inverted to make the pair inward facing. The Illumina paired-end library, constructed by using a PCR-free kit, was sequenced by MiSeq for 251 bp apiece from both ends, and we obtained 3.6 M paired-end (PE) reads. The reads were subjected to SRM trimming, in which 21-mers appearing fewer than three times were regarded as sequence errors or corresponding to adapter junctions. GS-FLX reads and MP and PE reads were used for assembly by Newbler version 2.8, in which 0.19 M FLX reads (121.9 Mb), 0.93 M MP reads (147 Mb), 3.3 M PE reads (598 Mb) were used (867 Mb in total), and we obtained 11 scaffolds and 167 contigs.

Finishing was conducted using GenoFinisher and AceFileViewer (8). The 58 repeat-induced gaps were closed by GenoFinisher and AceFileViewer. To close 13 gaps that arose by a lack of reads, the Illumina reads before trimming and the reads from mate-pair library that were not extracted as mate pairs were searched for reads that filled the gaps.

The complete sequence of the strain No. 7 genome comprised one circular chromosome of 4,599,046 bp and two linear plasmids of 135,111 bp and 93,808 bp. The linear plasmid ends were confirmed by examining MP reads that constituted the plasmid ends. We found that those reads before adapter trimming carried a single 19-nucleotide (nt) adapter of the Nextera mate-pair library (AGATGTGTATAAGAGACAG) joined to the end of the plasmids.

The finished sequence was confirmed by FinishChecker, annotated by the NCBI Prokaryotic Genomes Annotation Pipeline (PGAP), and curated using GenomeMatcher (9). While referring to annotation data obtained from the Microbial Genome Annotation Pipeline (http://www.migap.org/), we corrected the start codon positions and added genes that were missing in the PGAP annotation.

### Nucleotide sequence accession numbers.

The genome sequence of *Microbacterium* sp*.* No. 7 has been deposited in the NCBI under the accession numbers CP012697 to CP012699.
